# When is the best time to sample aquatic macroinvertebrates in ponds for biodiversity assessment?

**DOI:** 10.1007/s10661-016-5178-6

**Published:** 2016-02-26

**Authors:** M. J. Hill, C. D. Sayer, P. J. Wood

**Affiliations:** Centre for Hydrological and Ecosystem Science, Department of Geography, Loughborough University, Loughborough, LE11 3TU Leicestershire UK; Pond Restoration Research Group, Environmental Change Research Centre, Department of Geography, University College London, London, WC1E 6BT UK

**Keywords:** Pond survey, Monitoring, Seasonal variability, Lentic ecosystems, Species richness

## Abstract

Ponds are sites of high biodiversity and conservation value, yet there is little or no statutory monitoring of them across most of Europe. There are clear and standardised protocols for sampling aquatic macroinvertebrate communities in ponds, but the most suitable time(s) to undertake the survey(s) remains poorly specified. This paper examined the aquatic macroinvertebrate communities from 95 ponds within different land use types over three seasons (spring, summer and autumn) to determine the most appropriate time to undertake sampling to characterise biodiversity. The combined samples from all three seasons provided the most comprehensive record of the aquatic macroinvertebrate taxa recorded within ponds (alpha and gamma diversity). Samples collected during the autumn survey yielded significantly greater macroinvertebrate richness (76 % of the total diversity) than either spring or summer surveys. Macroinvertebrate diversity was greatest during autumn in meadow and agricultural ponds, but taxon richness among forest and urban ponds did not differ significantly temporally. The autumn survey provided the highest measures of richness for Coleoptera, Hemiptera and Odonata. However, richness of the aquatic insect order Trichoptera was highest in spring and lowest in autumn. The results illustrate that multiple surveys, covering more than one season, provide the most comprehensive representation of macroinvertebrate biodiversity. When sampling can only be undertaken on one occasion, the most appropriate time to undertake surveys to characterise the macroinvertebrate community biodiversity is during autumn, although this may need to be modified if other floral and faunal groups need to be incorporated into the sampling programme.

## Introduction

It is only relatively recently that ponds have been widely recognised as important freshwater habitats supporting aquatic biodiversity in Europe (Davies et al. 2008; Picazo et al. 2012; Hassall and Anderson 2015). In particular, ponds have often been shown to support higher numbers of rare and uncommon taxa than other freshwater habitats such as rivers and lakes (Williams et al. 2003; Biggs et al. 2005; Lukacs et al. 2013). The number of peer-reviewed, scientific publications examining pond biodiversity has tripled in the last decade (Céréghino et al. 2014), and a few key conservation project initiatives have elevated pond habitats and the organisms they support up the conservation agenda (e.g. Freshwater Habitats Trust 2015b, c; DCPWA 2015). Nonetheless, while legislation has necessitated the monitoring of larger freshwater bodies (rivers and lakes) at the European and national levels, following the adoption of the EU Water Framework Directive into law (EC 2000; Oertli et al. 2005; Birk et al. 2012), routine monitoring of small waterbodies such as ponds is rarely undertaken. As a result, research focused on the repeated monitoring of ponds and how best to achieve this is limited.

Ponds support a wide range of flora and fauna with highly variable life histories and habitat preferences that need to be considered when designing sampling programmes. If the primary focus of the pond survey is to sample aquatic macroinvertebrates, there are clear standardised protocols for sampling (e.g. the National Pond Survey; Biggs et al. 1998, Predictive SYstem for Multimetrics—PSYM; Environment Agency and Ponds Conservation Trust 2002; Chadd 2010). For macroinvertebrates, these almost exclusively involve the use of a ‘pond net’ and the application of a sweep sampling technique for a fixed/standardised time period (Oertli et al. 2005; Hassall and Anderson 2015) with sampling effort divided between different habitat units (Gioria et al. 2010; Becerra-Jurado et al. 2012). However, there are a number of specific variations and modifications to the protocol that can be used when sampling particular macroinvertebrate groups, such as Odonata (Oertli et al. 2005; Ruggiero et al. 2008; Raebel et al. 2011) and Chironomidae (Rufer and Ferrington 2008; Michelutti et al. 2011; Ruse 2013). Other protocols have been designed to cover multiple groups, for example, the European Plans d’eau Suisses (PLOCH) sampling methodology focusses on five target groups: aquatic macrophytes, Coleoptera, Odonata, Gastropoda and Amphibia. This methodology combines a fixed 3-min methodology for aquatic Coleoptera and Gastropoda with alternative sampling strategies for macrophytes, Amphibia and larval Odonata, to provide a rapid assessment of pond taxonomic richness (Oertli et al. 2005).

When attempting to characterise macroinvertebrate diversity, despite some standardised approaches to pond sample collection (PSYM and PLOCH methodologies), there is considerable variability in the timing of sampling across Europe. In general, academic studies reporting pond biodiversity have collected samples over a single sampling season, most frequently summer (e.g. Jeffries 1991; Biggs et al. 2007; Colding et al. 2009; Le Viol et al. 2009; Gioria et al. 2010; Sayer et al. 2012; Usio et al. 2013; Briers 2014; Noble and Hassall 2014). Indeed, the two principal methodologies for quantifying the ecological quality of ponds in the UK (PSYM) and Europe (PLOCH) both advocate summer sampling (Environment Agency and Ponds Conservation Trust 2002; Oertli et al. 2005). A number of published studies, on the other hand, have conducted sampling during either the spring or autumn seasons (*spring*—Collinson et al. 1995; Bazzanti et al. 2010; Fuentes-Rodríguez et al. 2013; Hassall and Anderson 2015; *autumn*—Brönmark 1985) or across two seasons (e.g. Wood et al. 2001; Della Bella et al. 2005; Declerck et al. 2006; Céréghino et al. 2008; Ruggiero et al. 2008; Becerra-Jurado et al. 2010; Nakanishi et al. 2014). Indeed, the UK national pond survey advocates that sampling should be undertaken over three seasons to obtain an accurate representation of total diversity (Biggs et al. 1998; Chadd 2010), and this has been implemented in some studies (e.g. Hill et al. 2015), while a small number of studies have even sampled aquatic macroinvertebrates on a monthly basis for a single year (e.g. Chaichana et al. 2011; Armitage et al. 2012), or in the case of ephemeral ponds to reflect the presence of water within the pond basin (Bilton et al. 2009; Florencio et al. 2009).

Given the variability in the season that pond macroinvertebrate surveys are undertaken, and to inform future studies of biodiversity assessment, the current study sought to (i) characterise the alpha and gamma diversity of aquatic macroinvertebrate communities for 95 ponds over three seasons (spring, summer and autumn) and (ii) examine the macroinvertebrate community heterogeneity (beta-diversity) among spring, summer and autumn seasons. Using data from 95 ponds, we examined how the timing of sample collection influenced measures of species diversity across an array of invertebrate groups to determine whether a single sampling period may be considered appropriate for assessments of biodiversity.

## Materials and methods

### Study sites

A total of 95 ponds within the catchment of the River Soar, close to the town of Loughborough (Leicestershire, UK), were sampled (68 perennial and 27 ephemeral ponds). The ponds were located in four land use types typical of a European lowland landscape: floodplain meadow (35 ponds), arable agricultural (12 ponds), deciduous forest (7 ponds) and urban environment (41 ponds). The latter group included ponds within domestic gardens, urban green spaces (such as parks) and highly developed areas (industrial, roadside and city centre) such as storm water-retention ponds.

### Aquatic macroinvertebrate sampling

Aquatic macroinvertebrate samples were collected on three occasions from each pond corresponding to spring (March), summer (June) and autumn (September) seasons. Not all ponds were wet on each sampling date; therefore, a total of 256 macroinvertebrate samples were collected (spring *n* = 84, summer *n* = 93 and autumn *n* = 79). In this study, a fixed-time macroinvertebrate sampling strategy (Biggs et al. 1998) was not deemed suitable for macroinvertebrate diversity assessment given the considerable seasonal variation in the wetted pond area (Armitage et al. 2012). To account for this variation, and to avoid any negative or destructive effects of sampling in very small waterbodies, the fixed-time sampling strategy was modified and the sampling time allocated to each pond was proportional to its surface area up to a maximum of 3 min (Biggs et al. 1998). Thus, ponds with a surface area >50 m^2^ were sampled for 3 min, while for smaller ponds, 30 s of sampling for every 10-m^2^ surface area was employed. A 1-mm-mesh standard pond net was used to sample aquatic macroinvertebrates. The total sampling time designated to each pond was divided equally between the habitat units present (e.g. emergent macrophytes, submerged macrophytes and open water). If one habitat type dominated, pond sampling time was divided to reflect this (Biggs et al. 1998). An inspection of any hard surfaces or larger substrates (e.g. large woody debris) for macroinvertebrate taxa was undertaken for up to 60 s during each sampling (Biggs et al. 1998). Sampling was not undertaken during the winter months as many aquatic invertebrates are relatively inactive due to reduced water temperatures, others may be present in the form of eggs or pupae which remain dormant until water temperatures increase in spring, while some adult life stages (e.g. Trichoptera and Coleoptera) seek refuge in adjacent terrestrial habitats (Chadd 2010), rendering them more difficult to sample. In addition, during winter, many floodplain ponds are inaccessible due to inundation by floodwaters. Aquatic macroinvertebrate samples from each season were preserved in the field and processed into 70 % industrial methylated spirit (IMS) prior to identification. Identification was undertaken to species level wherever possible; however, dipteran larvae and Planariidae were identified to family level and Hydrachnidiae, Oligochaeta and Collembola were recorded as such.

### Statistical analyses

Aquatic macroinvertebrate diversity was examined across the three sampling seasons (spring, summer and autumn) by combining habitat species-abundance data for each site for all seasons. Macroinvertebrate community abundance and alpha diversity (characterised by taxon richness, the Shannon-Wiener diversity index and the Berger-Parker dominance index) were calculated for each pond site in each season using Species Diversity and Richness IV software (Pisces Conservation 2008). Prior to statistical analysis, the data was examined to ensure compliance with the underlying assumptions of parametric tests (e.g. normal distributions). Where data violated these assumptions (e.g. abundance data), they were log_10_ transformed. The statistical significance of variance in pond taxon richness, abundance, the Shannon-Wiener diversity index and the Berger-Parker dominance index between spring, summer and autumn seasons among the four pond types was examined using nested analysis of variance (season nested within pond type) (Van de Meutter et al. 2005). The statistical significance of differences between the main macroinvertebrate groups and season was examined using one-way ANOVA. A post hoc Sidak test was employed to determine where significant differences between seasons occurred. All univariate analyses were undertaken in IBM SPSS Statistics (version 21, IBM Corporation, New York). The heterogeneity of seasonal macroinvertebrate communities (beta-diversity) was examined using analysis of similarity (ANOSIM) and non-metric multidimensional scaling (NMDS—using Bray-Curtis dissimilarity metric), undertaken using PRIMER 6 (Clarke and Gorley 2006). Species-abundance data were log (*X* + 1) transformed prior to ANOSIM and NMDS analysis.

## Results and discussion

### Macroinvertebrate diversity

A total of 228 taxa were recorded from 95 ponds over the three seasons, representing 19 orders and 68 families (Table 1). Sampling across all three seasons provided the greatest aquatic macroinvertebrate biodiversity for the ponds examined. In addition, the inclusion of data from surveys for multiple seasons clearly provided greater detail on the composition of the invertebrate community and by extension an improved basis for management/conservation strategies designed to enhance pond biodiversity. However, undertaking surveys over three seasons raises a number of practical considerations in relation to financial cost and the time required to collect, process and identify samples, especially when stakeholders have limited resources and rapid delivery of project results is required (Oertli et al. 2005). This is especially true of pond restoration studies, where a minimum of 2–3 years of sampling is required to determine if restoration measures have been successful (e.g. Sayer et al. 2013). In addition, many large-scale pond surveys rely on volunteers/citizen scientists to undertake the sampling (Freshwater Habitats Trust 2015a) and the requirement for samples over more than one season may discourage volunteers from participating due to the increased time commitment. As a consequence, sampling of ponds has typically been undertaken over one season by necessity; this raises the question as to the optimum time to collect samples for biodiversity assessment.

**Table 1:** Summary table of the number of taxa and abundance of macroinvertebrates collected from the three sampling seasons: spring 2012, summer 2012 and autumn 2012

	Spring	Summer	Autumn	Total (all seasons combined)
Total taxon richness	166	154	174	228
Mean taxon richness	14	14	22	29
Mean abundance	538	498	1185	1948
% of total taxon richness (all seasons combined) supported	72 %	68 %	76 %	100 %

**Fig. 1:**
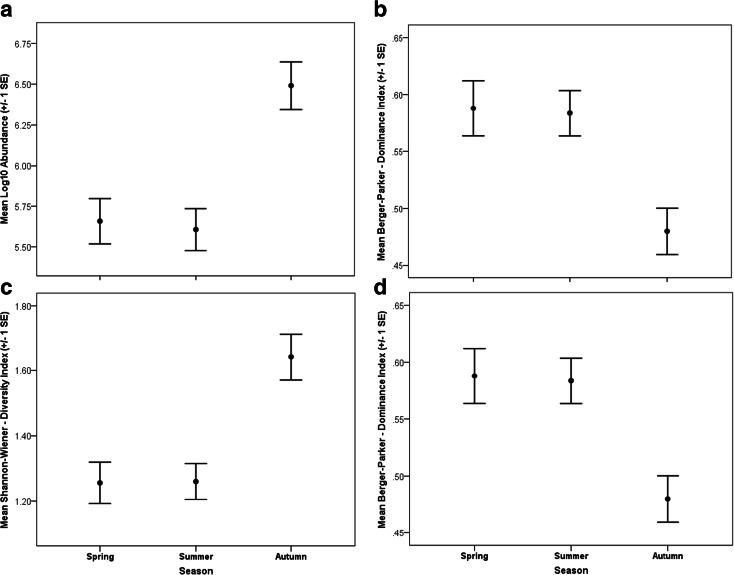
Mean (±1 SE) community abundance (log_10_) (**a**), taxon richness (**b**), Shannon-Wiener diversity index (**c**) and Berger-Parker dominance index (**d**) recorded for ponds during the spring, summer and autumn sampling seasons

If pond surveys are by necessity restricted to a single season, due to time and financial constraints, the results of this study indicate that the autumn (Sept–Oct) period yields the greatest macroinvertebrate biodiversity and supports the findings reported by Chadd (2010). Significantly greater taxon richness (ANOVA *F*_2, 255_ = 9.760; *p* < 0.01), macroinvertebrate abundance (ANOVA *F*_2, 255_ = 7.284; *p* < 0.01) and Shannon-Wiener diversity index scores (ANOVA *F*_2, 255_ = 5.139; *p* < 0.01) were recorded from ponds (alpha diversity) during autumn compared to spring and summer seasons (Fig. 1; Table 1). Some 76 % of total macroinvertebrate richness (174 taxa) was recorded in the autumn survey (228 taxa for all three seasons—Table 1). Further, the Berger-Parker dominance index was significantly lower (ANOVA *F*_2, 255_ = 3.236; *p* < 0.01) in autumn compared to that in spring and summer (Fig. 1). Similar autumn peaks in macroinvertebrate biodiversity have been recorded in other studies in the UK, covering a range of pond types and settings, suggesting consistent seasonal patterns (Wood et al. 2001; Armitage et al. 2012). Pond restoration involving scrub and sediment removal is typically undertaken during early autumn after amphibian juveniles have migrated away from the pond basin and when farmland birds have finished rearing young. Thus, one advantage of autumn sampling is that it can be undertaken just prior to restoration management activities (Sayer et al. 2013). While the autumn season may be the optimal sampling period for ponds in lowland temperate maritime regions of Northern Europe and North America, it should be noted that the best time to sample pond communities in arid, semi-arid Mediterranean, tropical/sub-tropical or polar climates will probably differ. Indeed, this is especially true of temporary ponds in drier climates, where diversity typically peaks in late spring and ponds are generally subject to drying and desiccation by mid-summer (Waterkeyn et al. 2008; Florencio et al. 2009, 2014; Díaz-Paniagua et al. 2010). Clearly, given the variable climate, hydrological regimes and invertebrate communities across different biomes, further research is required to determine the most appropriate time to sample macroinvertebrate biodiversity.

**Fig. 2:**
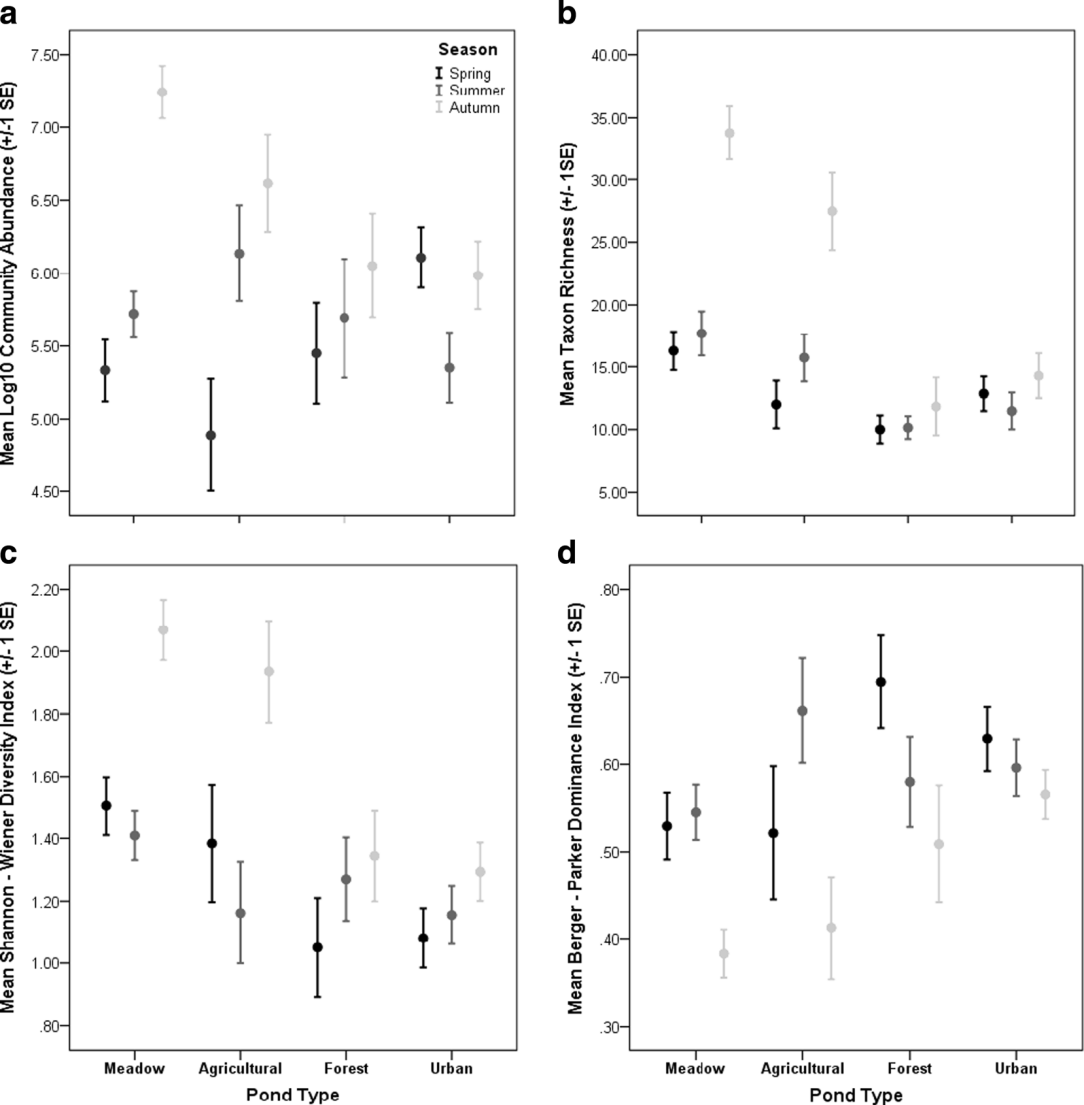
Mean (±1 SE) community abundance (log_10_) (**a**), taxon richness (**b**), Shannon-Wiener diversity index (**c**) and Berger-Parker dominance index (**d**) recorded for meadow, agricultural, forest and urban ponds during the spring, summer and autumn sampling seasons

**Fig. 3:**
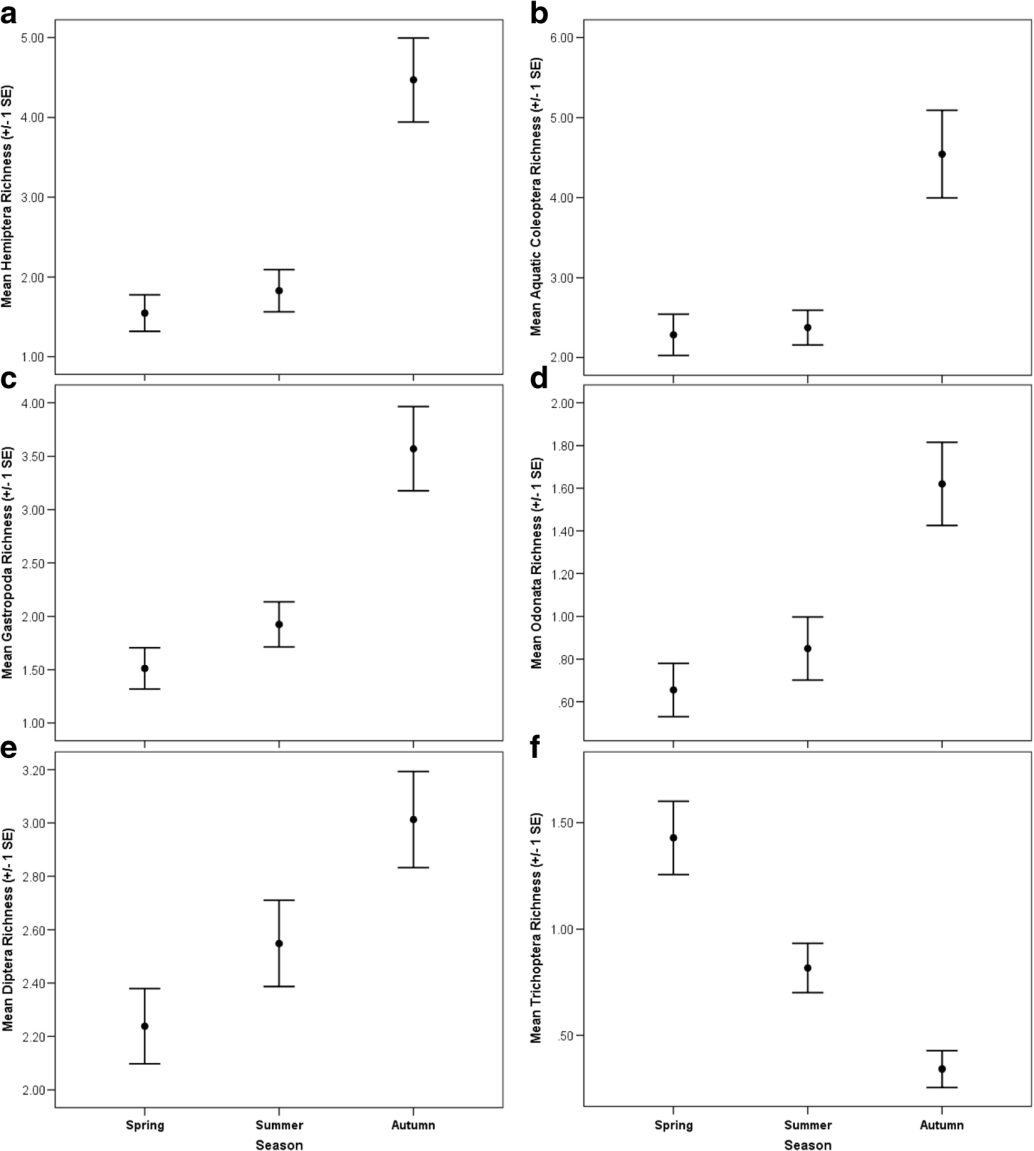
Mean (±1 SE) taxon richness of Hemiptera (**a**), aquatic Coleoptera (**b**), Gastropoda (**c**), Odonata (**d**), Diptera (**e**) and Trichoptera (**f**) recorded for ponds during the spring, summer and autumn sampling seasons

In this study, some inconsistencies were evident in terms of macroinvertebrate seasonal responses across different land uses. Community abundance increased seasonally from spring to autumn in meadow, agricultural and forest ponds, but within urban ponds, abundance was lower during summer (Fig. 2). Macroinvertebrate richness and Shannon-Wiener diversity index scores were highest during autumn compared to those during spring and summer among meadow and agricultural ponds, but were not significantly different among seasons for forest and urban ponds (Fig. 2). Nonetheless, the Berger-Parker dominance index was lowest in autumn in all four pond types (Fig. 2). For alpha diversity, a significantly greater diversity of Hemiptera (ANOVA *F*_2, 255_ = 20.057; *p* < 0.001), aquatic Coleoptera (particularly Dytiscidae) (ANOVA *F*_2, 255_ = 12.423; *p* < 0.001), Gastropoda (ANOVA *F*_2, 255_ = 15.220; *p* < 0.001) and Odonata (ANOVA *F*_2, 255_ = 10.085; *p* < 0.001) taxa was recorded during autumn compared to spring and summer (Fig. 3a–d). Additionally, significantly greater diversities of Diptera (ANOVA *F*_2, 255_ = 5.542; *p* < 0.005) were recorded in the autumn compared to the summer season (ANOVA *p* < 0.05) (Fig. 3e). In contrast, Trichoptera (particularly the families Limnephilidae and Leptoceridae) was characterised by significant reductions in taxon richness during the autumn season (ANOVA *F*_2, 255_ = 16.575; *p* < 0.001) (Fig. 3f). Species within these trichopteran families typically emerge as adults during summer and autumn (Wallace et al. 2003), greatly reducing their abundance and diversity when compared to spring. Similar patterns may also occur for other univoltine aquatic insect orders such as Ephemeroptera and Plecoptera with life histories including aerial dispersal and reproductive phases (Menetrey et al. 2008, 2011), although both orders did not constitute major components of abundance or biodiversity (eight taxa) in this study.

### Pond community heterogeneity across different land uses

**Fig. 4:**
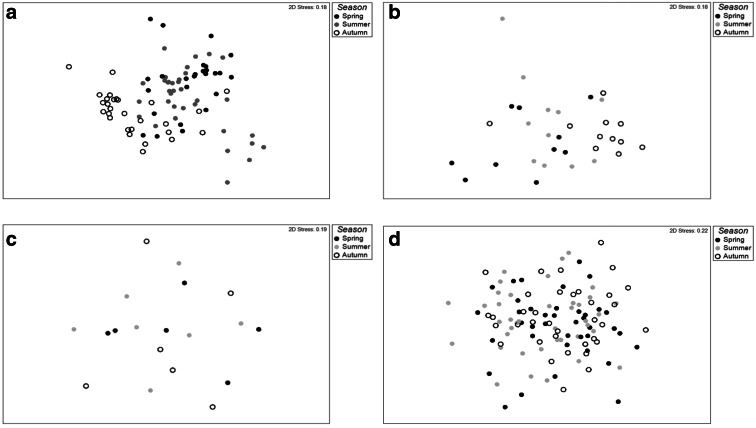
Two-dimensional NMDS plot of dissimilarity (Bray-Curtis) of seasonal (spring, summer and autumn) invertebrate communities within the four pond types; **a** meadow, **b** agricultural, **c** forest and **d** urban

Significant macroinvertebrate community heterogeneity (beta-diversity) was recorded between the autumn season and the other two seasons (spring and summer) among the meadow and agricultural ponds (ANOSIM *p* < 0.005). In addition, macroinvertebrate community composition within meadow ponds during spring was significantly different compared to that during summer. This distinction between autumn invertebrate communities and other seasons for the meadow and agricultural ponds is clearly demonstrated in the NMDS plots (Fig. 4a, b). In marked contrast, no significant seasonal difference in macroinvertebrate community heterogeneity was observed for the forest and urban ponds (ANOSIM *p* > 0.05) as illustrated by overlap of samples in the NMDS plots for all three seasons (Fig. 4c, d). The open landscape associated with meadow and agricultural ponds may have enabled macroinvertebrate taxa to disperse and colonise other ponds more easily, which in turn may have facilitated the clear seasonal succession of taxa. In contrast, for urban and forest ponds, there was little seasonal difference in community composition or biodiversity. This probably reflects the structure of urban and forest landscapes. In urban areas, physical structures and management regimes may limit dispersal potential (active and passive) between ponds (Fahrig 2003), resulting in reduced opportunities for the recruitment of new invertebrate taxa. However, the similar faunal community composition recorded over the three seasons within urban ponds may also reflect the harsh environmental conditions generally associated with the urban environment, especially reduced refugia in urban ponds as a result of lower macrophyte coverage, reduced water quality from urban runoff, high densities of benthivorous fish and the non-natural bank (Heal et al. 2006; Hassall 2014; Hassall and Anderson 2015).

The long-term conservation of pond habitats is typically based on the presence of rare and endangered taxa and/or very high biodiversity (Hassall et al. 2012). For example, in the UK, the designation of a pond as a Priority Habitat under the UK Post-2010 Biodiversity Framework (previously the biodiversity action plan) requires ponds to support >50 aquatic macroinvertebrate taxa, Red Data Book species, UK Biodiversity Action Plan species or 3 nationally scarce aquatic macroinvertebrate taxa (BRIG 2008; JNCC 2012). Based on the results of this study, sampling over three seasons or, if restricted to one season, during autumn clearly provides the best opportunity to capture the greatest aquatic macroinvertebrate biodiversity in ponds. Currently, the most widely employed methodologies for sampling ponds across Europe are based on summer surveys reflecting the desire to sample multiple groups of organisms, including littoral and aquatic macrophytes, macroinvertebrates, amphibians and fish (Environment Agency and Ponds Conservation Trust 2002; Oertli et al. 2005). However, single-season sampling will result in the underestimation of biodiversity of one or more of the groups. As a result, it is important to clearly define the primary purpose of the sampling programme and its potential limitations in terms of the flora and fauna examined. Based on the results of this study, an overview of the ‘best’ season for aquatic macroinvertebrate surveys, which reflects the natural heterogeneity of the different groups and land use, can be made (Table 2). We recognise that this assessment may be incomplete and that in other biogeographical regions subject to different hydro-climatological regimes, additional surveys timed to coincide with particular life history stages may be required, with this especially true of rare or endangered species. In addition, for other taxonomic groups within ponds, it may be appropriate or necessary to sample at other times. For example, amphibians are usually sampled during spring and/or early summer to assess breeding success and to capture various life stages prior to their seasonal dispersal into the wider environment (Rubbo and Kiesecker 2005). Sampling of macrophytes is typically undertaken during the summer or early autumn months, when aquatic vegetation is more readily identifiable due to the presence of flowers and fruiting bodies (Akasaka and Takamura 2012) and dragonflies can also be effectively recorded during this time window. This study clearly illustrates that for aquatic macroinvertebrates the timing of the survey(s) depends on the purpose and information required and that multiple surveys in a single year provide the most comprehensive picture of total biodiversity. However, targeted surveys form an essential part of contemporary conservation and a balance is required between economic reality, scientific needs and a desire for data to underpin on-going management activities. Given the significant biological diversity and conservation value of ponds (Davies et al. 2008; Céréghino et al. 2014) and the services they provide to humans (e.g. diffuse pollutant removal, carbon sequestering, flood reduction and water collection; Downing et al. 2008; Céréghino et al. 2014), statutory monitoring of these small freshwater habitats would be desirable to ensure the persistence and survival of freshwater biota in urban and rural areas and to assess the success of conservation efforts and restoration projects.

**Table 2:** Proposed best time to sample total macroinvertebrate diversity and particular macroinvertebrate groups if restricted to a single survey season across four land use types

	Total diversity	Coleoptera	Hemiptera	Gastropoda	Odonata	Diptera	Trichoptera
Landscape	Autumn	Autumn	Autumn	Autumn	Autumn	Autumn	Spring
Meadow	Autumn	Autumn	Autumn	Autumn	Autumn	Autumn	Spring
Agricultural	Autumn	Autumn	Autumn	Autumn	Autumn	Autumn	Spring or summer
Forest	Any	Autumn	Any	Any	Any	Summer or autumn	Spring
Urban	Any	Any	Autumn	Any	Autumn	Any	Spring

## Summary and conclusions

A total of 95 ponds were used to examine the taxonomic richness recorded from aquatic macroinvertebrate pond surveys across three seasons. The results of this study demonstrate that surveying aquatic macroinvertebrate communities across three seasons provides the most accurate representation of aquatic macroinvertebrate biodiversity within pond habitats, compared to single-season sampling. Indeed, restricting aquatic macroinvertebrate surveys to a single season may lead to major underrepresentation of total biodiversity. However, if surveys are confined to a single season, the results of this study indicate that autumn sampling provides the best opportunity for the evaluation of total macroinvertebrate biodiversity. Determining which season(s) provides the most comprehensive representation of aquatic macroinvertebrate biodiversity in ponds can provide more accurate information for the development and implementation of conservation and management strategies of ponds and the communities they support.
